# Fusion Methods for Biosignal Analysis: Theory and Applications

**DOI:** 10.1155/2017/7152546

**Published:** 2017-11-19

**Authors:** Addisson Salazar, Vicente Zarzoso, Manuel Rosa-Zurera, Luis Vergara

**Affiliations:** ^1^Institute of Telecommunications and Multimedia Applications, Universitat Politècnica de València, Camino de Vera, s/n, 46022 Valencia, Spain; ^2^I3S Laboratory, Université Côte d'Azur, CNRS, Sophia Antipolis, France; ^3^Universidad de Alcalá, Campus Universitario, Alcalá de Henares, Spain

Recent advances in data acquisition and biosignal processing are paving the way for the optimal integration or fusion of complementary data modalities in a wide variety of clinical settings. Data modalities include electrocardiography (ECG), electroencephalography (EEG), electrocorticography (ECoG), magnetic resonance imaging (MRI), functional MRI (fMRI), positron emission tomography (PET), and diffusion tensor imaging (DTI). Integration can be performed by exploiting the analyses sequentially or simultaneously, depending on issues related to synchronization, physical compatibilities, and standard clinical procedures. Fusion approaches aim at integrating analyses of data from different modalities, establishing synergic relationships for improved clinical hypothesis testing and medical diagnosis.

The heterogeneous nature of data sources from different clinical analyses and acquisition modalities presents big challenges. The main objective of data fusion is to exploit complementary properties of several single-modality methods in order to improve each of them considered separately. In addition, fusion can enable or enhance the approximation to more complex structured results (e.g., hierarchical trees and topological networks). This broad field of research has been named in different ways, for instance, sensor data fusion, decision fusion, multimodal fusion, heterogeneous sensor fusion, mixture of experts, classifier combiners, and multiway signal processing.

This special issue focuses on theoretical and application advances in fusion methods for biosignal analysis (FMBA). Fourteen submissions were received and each manuscript was reviewed by at least two external referees. Five papers were finally accepted for the special issue. The accepted papers cover important problems related to computational intelligence and neuroscience.

In one of the papers, H. Banville et al. propose a method for mental task evaluation based on the fusion of features extracted from EEG and near-infrared spectroscopy. The tasks evaluated are word generation, mental rotation, subtraction, singing and navigation, and motor and face imagery. The method is intended to improve classification performance for more efficient and flexible brain computer interfaces (BCIs).

Y. Huang et al. develop a multimodal late fusion procedure that combines features from facial expression images and EEG data for emotion recognition. A two decision-level fusion is performed to classify four basic emotion states (happiness, neutral, sadness, and fear) and three emotion intensity levels (strong, ordinary, and weak).

A general-purpose method for classification named dandelion algorithm (DA) is developed in the paper by X. Li et al. Different combinations of DA with other bioinspired methods (bat algorithm, particle swarm optimization, and enhanced fireworks algorithm) and a neural network-based method called extreme learning machine are implemented. Results show improvement in classification accuracy for different biomedical problems using data from EEG, ECG, and single photon emission computed tomography (SPECT).

R. Liu et al. put forward a method to classify anisomerous motor imagery states using EEG sensors for BCI systems. The states consist of imaginary movements with the left hand, right foot, and right shoulder, as well as the resting state. The fusion is performed using weights obtained by *k*-nearest neighbour and support vector machine classifiers.

Finally, Z. Weng et al. design a multimodal fusion method for localization of hub regions in the brain using MRI T1 and DTI. The number of streamlines is fused with normalized fractional anisotropy for more comprehensive brain bioinformation. The importance value of each node and hub localization is estimated by combining the brain region importance contribution matrix and the information transfer efficiency value. The method is applied to schizophrenia analysis.

The above papers form a motivating sample of the real problems and solutions that can be approached from the perspective of FMBA including theory and applications. The relevance of the topic is shown by the significant improvements obtained over competing approaches in the different contexts. We hope that readers will find insights and inspiration for further research on FMBA.

